# To Dr. Bradley

**Published:** 1802-05

**Authors:** 


### To Dr. BRADLEY.

Sir,

As the following inſtance of Quack credulity came to my knowledge only two days ago, I will thank you, *if approved of*, to inſert it in the Paper on Quackery, ſigned Medicus, either as a Note, or otherwiſe.

Leſt, however, you ſuſpect ſome improper conduct in this anonymous Communication, ſimilar to what I am about to mention, I ſhall ſign my name, which, however, on the preſent ſubject, I do not wiſh to have publiſhed. The character of Mr. Robert Booby, in the paper alluded to, you may perhaps object to, as not knowing whether it be a real name or fictitious; but had it been the real name, the individual is ſo obſcure, and lives in ſo obſcure a part of the country, that it would be ſcarcely poſſible to diſcover the prototype.

The following may ſerve as an inſtance of the credit due to Quack Advertiſements; "About a year and a half ago an advertiſement appeared in the public papers, ſigned, to the beſt of my recollection, Richard Maſtyn, Hedley Hall, Northumberland, ſtating, that the writer's daughter had been for a long time in a moſt deplorable ſtate, for which the moſt eminent of the Faculty in Newcaſtle had been conſulted in vain. Very fortunately fortunately, however, the father was induced to try the celebrated brown and yellow worm lozenges, which, wonderful to relate, reftored the child to perfect health in a fhort time. The affectionate father, delighted with having a darling child fnatched from the brink of the grave, of courfe confidered it as a duty to make the cure thus public.

This handfome acknowledgement gratified the patentee fo highly, that it was foifted into moft of the public papers for a length of time, until the writer declared he was heartily fick with feeing it.

Unfortunately, however, this flaming epiftle proved to be merely a *waggifh trick*, intended to tickle the ear of this difciple of Con-fu-tfi; a fpecies of luxury which the ingenious writer knew was fo peculiarly grateful to his countrymen. Thus, a juvenile frolic, one of thofe giddy tricks which we are all too apt to commit en gaieté de cœur, was eagerly caught at, and ferved to place, very unintentionally, one of the brighteft gems in the cap of this powerful Mandarin. Were medical men to be more ftrict in their refearches, they might eafily difcover feveral fuch pious frauds, and by degrees "ftrip the daw of his borrowed plumes."

*South Shields, April* 11, 1802.

